# Deep septal pacing and subsequent individualized interventricular electrical optimization in HCM with preserved ejection fraction and high anticipated pacing burden: a case report

**DOI:** 10.3389/fcvm.2026.1948665

**Published:** 2026-09-08

**Authors:** Ali Hakan Konuş

**Affiliations:** Department of Cardiology, Bingöl State Hospital, Bingöl, Türkiye

**Keywords:** cardiac physiologic pacing, conduction system pacing, deep septal pacing, hypertrophic cardiomyopathy, implantable cardioverter-defibrillator, interventricular electrical optimization, right bundle branch block

## Abstract

Management of patients with hypertrophic cardiomyopathy (HCM), preserved left ventricular ejection fraction, and high anticipated ventricular pacing burden remains challenging because current pacing guidelines provide limited direction for this clinical scenario. A 68-year-old man with non-obstructive HCM, symptomatic 2:1 atrioventricular block, and nonsustained ventricular tachycardia underwent implantation of a cardiac resynchronization therapy–defibrillator (CRT-D) system using a lumenless septal pacing lead connected to the LV port. Lead implantation proved technically challenging owing to marked septal hypertrophy (31 mm) with increased tissue resistance during lead advancement; despite repeated attempts at different septal sites, further lead advancement could not be achieved, and the final lead position remained within the deep interventricular septum. Stepwise lead advancement resulted in progressive shortening of the V6 R-wave peak time from 114 ms to 87 ms and paced QRS narrowing from 168 ms to 143 ms with deep septal pacing alone. Although the clinical trajectory was favorable, electrical assessment at the two-week follow-up demonstrated a residual 39-ms V6-to-V1 activation delay with deep septal pacing alone. Individualized ECG-guided VV optimization was therefore performed using coordinated deep septal and right ventricular pacing, bringing V6-to-V1 activation timing toward near-equalization with septal pre-excitation of 20 ms and further reducing QRS duration from 143 ms to 134 ms. At one month, NYHA functional class improved from III to I–II and NT-proBNP decreased from 1,868 to 369 pg/mL, with both remaining stable at one year. The clinical improvement was likely multifactorial; the specific contribution of VV optimization to this outcome could not be determined from a single case. This case highlights the potential value of device configurations that enable systematic post-implant interventricular electrical optimization as part of individualized programming in patients with structurally complex substrates and high anticipated pacing burden.

## Introduction

Hypertrophic cardiomyopathy (HCM) is a structurally and electrically heterogeneous disease that may be complicated by conduction abnormalities and ventricular arrhythmias (1). In patients who develop high-grade atrioventricular (AV) block, permanent pacing becomes necessary; however, the optimal pacing strategy remains uncertain, particularly in the presence of preserved left ventricular ejection fraction (LVEF). Conventional right ventricular pacing may exacerbate ventricular dyssynchrony, while cardiac resynchronization therapy (CRT) is generally reserved for patients with reduced LVEF and established dyssynchrony, leaving a clinically relevant gap in those with preserved systolic function but high anticipated pacing burden (2). Conduction system pacing, including left bundle branch area pacing, has emerged as a physiological alternative aimed at preserving native ventricular activation (3). Although its role is increasingly recognized, data remain limited in patients with HCM, particularly in complex clinical scenarios involving coexisting conduction disease and ventricular arrhythmias requiring implantable cardioverter-defibrillator (ICD) therapy, where both device selection and optimization of ventricular activation may be challenging. In such patients, the choice of pacing modality may carry important hemodynamic implications despite the absence of conventional CRT indications. Here, we report a case of hypertrophic cardiomyopathy with preserved LVEF, high-grade atrioventricular block, and nonsustained ventricular tachycardia in which individualized ECG-guided interventricular electrical optimization was used after deep septal pacing to address residual interventricular activation delay.

## Case presentation

A 68-year-old man with a known history of non-obstructive hypertrophic cardiomyopathy (HCM) was followed in the cardiology clinic. He also had a history of non-obstructive coronary artery disease previously documented by coronary angiography, and was receiving acetylsalicylic acid (100 mg daily) and atorvastatin (40 mg daily). There was no family history of sudden cardiac death. He presented with palpitations, intermittent dizziness, and exertional dyspnea on minimal activity [New York Heart Association (NYHA) class III].

**Figure 1:**
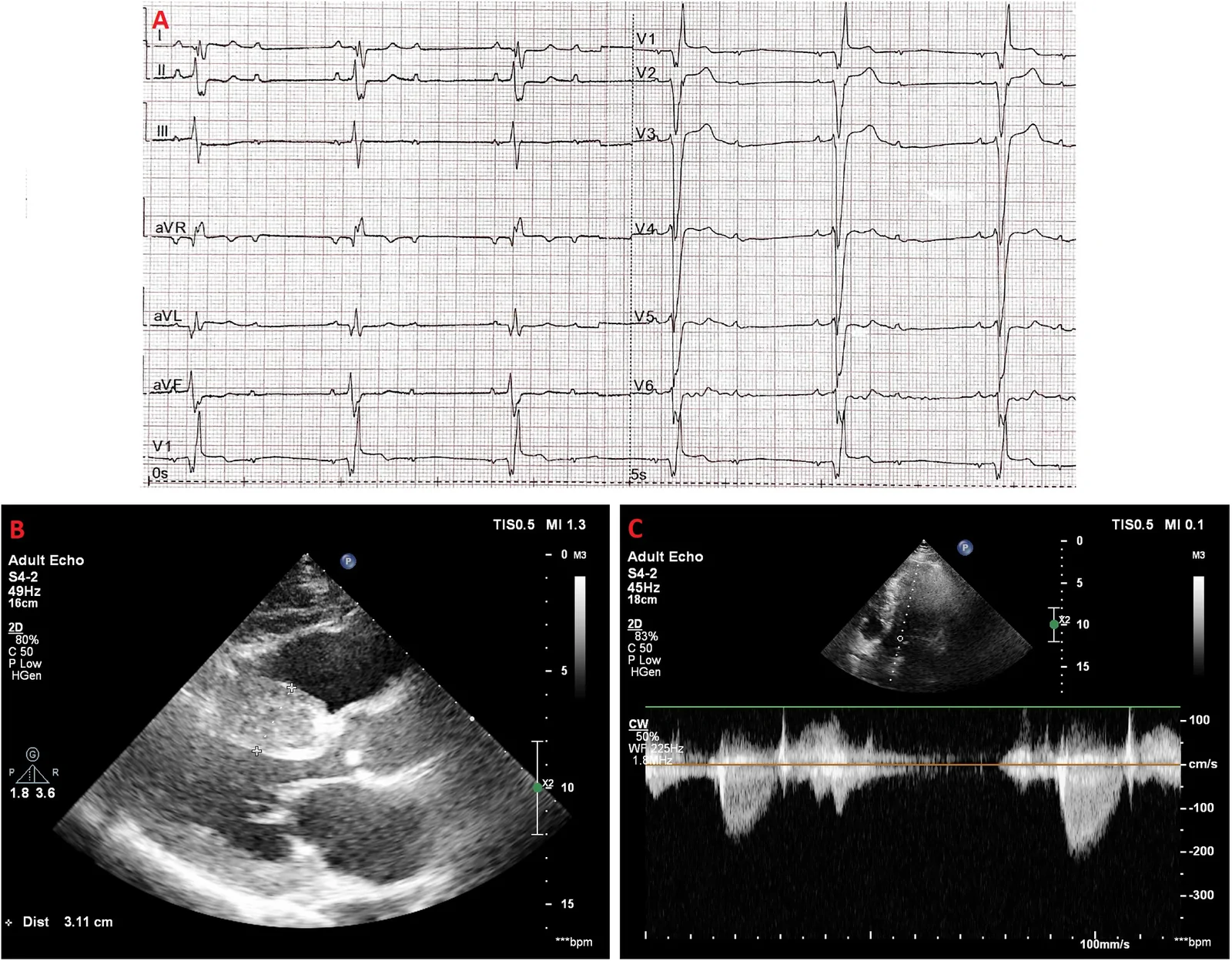
Baseline electrocardiographic and echocardiographic characteristics. **(A)** Twelve-lead electrocardiogram demonstrating sinus rhythm with 2:1 atrioventricular block (ventricular rate approximately 35 bpm), right bundle branch block morphology, QRS fragmentation and notching in multiple limb and precordial leads, and a qR pattern in lead V1. The baseline QRS duration was 168 ms. **(B)** Parasternal long-axis echocardiographic view showing marked asymmetric septal hypertrophy with an interventricular septal thickness of 31 mm. **(C)** Continuous-wave Doppler interrogation of the left ventricular outflow tract demonstrating the absence of significant left ventricular outflow tract obstruction at rest, consistent with non-obstructive hypertrophic cardiomyopathy.

Baseline electrocardiography demonstrated sinus rhythm with 2:1 atrioventricular (AV) block (ventricular rate of approximately 35 bpm) and a right bundle branch block morphology with a QRS duration of 168 ms, with QRS fragmentation and notching observed in multiple limb and precordial leads. The frontal QRS axis was approximately 0°, with positive deflection in lead I and near-isoelectric configuration in the inferior leads. A qR pattern was observed in lead V1, accompanied by mild ST-segment elevation in V1 (Figure 1A).

Transthoracic echocardiography revealed preserved left ventricular ejection fraction (LVEF 60%) with marked asymmetric septal hypertrophy (interventricular septum thickness 31 mm) (Figure 1B). No significant left ventricular outflow tract (LVOT) obstruction was detected at rest or with Valsalva maneuver (Figure 1C). Trace tricuspid regurgitation was present at baseline. Baseline NT-proBNP level was 1,868 pg/mL.

Twenty-four-hour ambulatory rhythm monitoring demonstrated persistent 2:1 AV block as the baseline rhythm, along with two episodes of monomorphic nonsustained ventricular tachycardia lasting 15–20 beats.

Given the coexistence of symptomatic high-grade AV block requiring permanent pacing and documented nonsustained ventricular tachycardia, combined device therapy incorporating both pacing and defibrillation capability was planned. Considering the preserved LVEF and absence of conventional indications for cardiac resynchronization therapy (CRT), a conduction system pacing–based strategy was preferred.

A cardiac resynchronization therapy–defibrillator (CRT-D) platform (Medtronic Compia MRI SureScan) was implanted, with a lumenless septal pacing lead (Medtronic 3830, 69 cm) connected to the LV port and the right ventricular lead connected to the RV port for defibrillation, sensing, and backup ventricular pacing. The lumenless lead was selected based on the operator's established experience with septal pacing and its controlled delivery characteristics, particularly in a patient in whom two transvenous leads were required to cross the tricuspid valve. At the time of implantation, an ICD-capable conduction system pacing lead (OmniaSecure) was not available in our practice. Although stylet-driven leads have also been used for conduction system pacing in patients with HCM, no specific lead design has been established as preferable for this clinical scenario.

**Figure 2:**
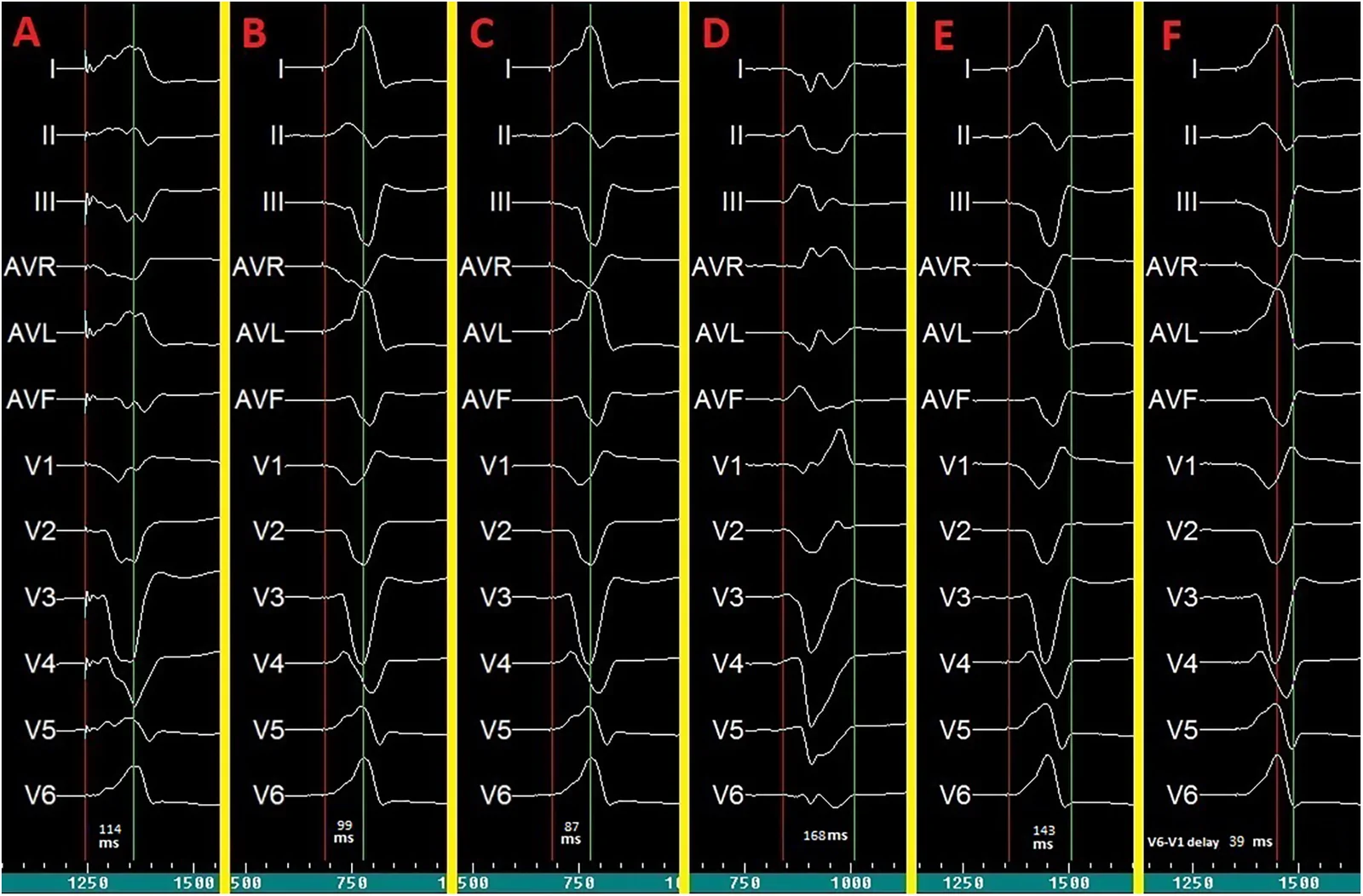
Electrocardiographic characterization of incremental lead advancement. **(A–C)** Serial surface ECG tracings obtained during stepwise advancement of the septal pacing lead, demonstrating progressive shortening of the V6 R-wave peak time (RWPT) from 114 ms at the initial septal position **(A)** to 99 ms at an intermediate position **(B)** and 87 ms at the final lead position **(C)**. **(D)** Surface ECG tracing at the time of implantation demonstrating the baseline QRS duration of 168 ms in the setting of right bundle branch block and 2:1 atrioventricular block. **(E)** Surface ECG tracing following final lead advancement demonstrating paced QRS narrowing to 143 ms. **(F)** Measurement of the V6-to-V1 R-wave peak time difference during deep septal pacing, showing a V6-to-V1 R-wave peak time difference of 39 ms. All paced QRS durations were measured from the pacing stimulus artifact to QRS offset. R-wave peak times were measured from the pacing stimulus artifact to the peak of the R wave in the respective lead.

Lead implantation was technically challenging because of marked septal hypertrophy (31 mm), with increased tissue resistance during lead advancement. Despite repeated attempts at different septal sites, deeper lead advancement could not be achieved, and progressive deep septal lead positioning resulted in stepwise improvement in electrical parameters. The V6 R-wave peak time (RWPT) decreased from 114 ms at the initial position to 99 ms at an intermediate position and ultimately to 87 ms at the final lead position (Figures 2A–C). A V6-to-V1 RWPT difference of 39 ms was observed at the final position, indicating earlier left ventricular activation (Figure 2F). Left ventricular activation time remained stable at both high- and low-output pacing; specifically, high-output pacing (5.0 V at 0.4 ms) and low-output pacing (0.5 V at 0.4 ms) demonstrated identical left ventricular activation times without significant change in V6 R-wave peak time. Stimulus-to-LVAT measurements were systematically assessed during output testing and showed no reproducible change suggestive of a transition between myocardial and conduction-system capture. No reproducible QRS morphology transition was observed with changes in pacing output. The paced QRS demonstrated a relatively gradual initial upstroke, suggesting a substantial myocardial activation component. The paced QRS morphology in lead V1 transitioned from the native qR pattern to a Qr configuration, with a smaller terminal R wave than at baseline.

**Figure 3:**
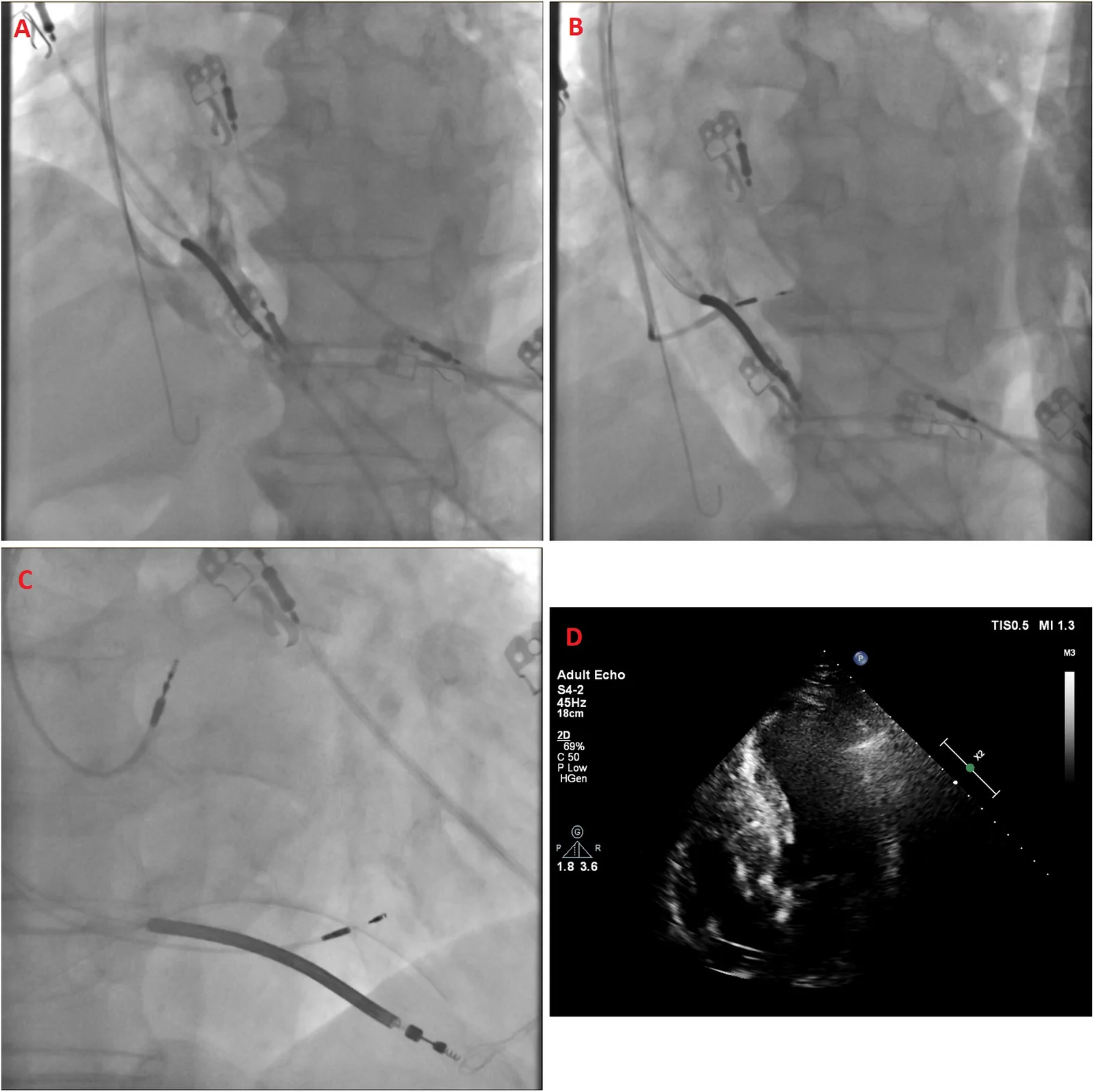
Fluoroscopic and echocardiographic assessment of septal lead implantation. **(A)** LAO 30° fluoroscopic projection demonstrating sheath positioning at the intended septal implantation site before lead advancement. **(B)** Contrast-enhanced fluoroscopic imaging demonstrating apparent intraseptal lead advancement. **(C)** RAO 30° fluoroscopic projection demonstrating the final lead trajectory and position. **(D)** Apical four-chamber echocardiographic view demonstrating stable intraseptal lead position within the markedly hypertrophied interventricular septum.

Fluoroscopic assessment demonstrated appropriate sheath positioning at the intended septal implantation site, progressive intraseptal lead advancement confirmed by contrast injection, and a stable final lead trajectory in both left and right anterior oblique projections (Figures 3A–D). Post-procedural electrocardiography demonstrated consistent ventricular capture with pacing stimuli preceding each QRS complex, a reduction in QRS duration from 168 ms to 143 ms, and a change in the frontal QRS axis (Figure 2E).

Pacing parameters were stable, with a capture threshold of 0.5 V at 0.4 ms, R-wave sensing of 17.4 mV, and satisfactory lead impedance. The ICD was programmed with standard ventricular tachycardia and ventricular fibrillation detection zones according to institutional practice, with a monitor zone configured for the nonsustained ventricular tachycardia range. The procedure was completed without acute complications.

Metoprolol was initiated following device implantation for arrhythmic prophylaxis and rate control; no additional heart failure–specific therapy was introduced, and the patient’s prior medical therapy was otherwise continued unchanged.

**Figure 4:**
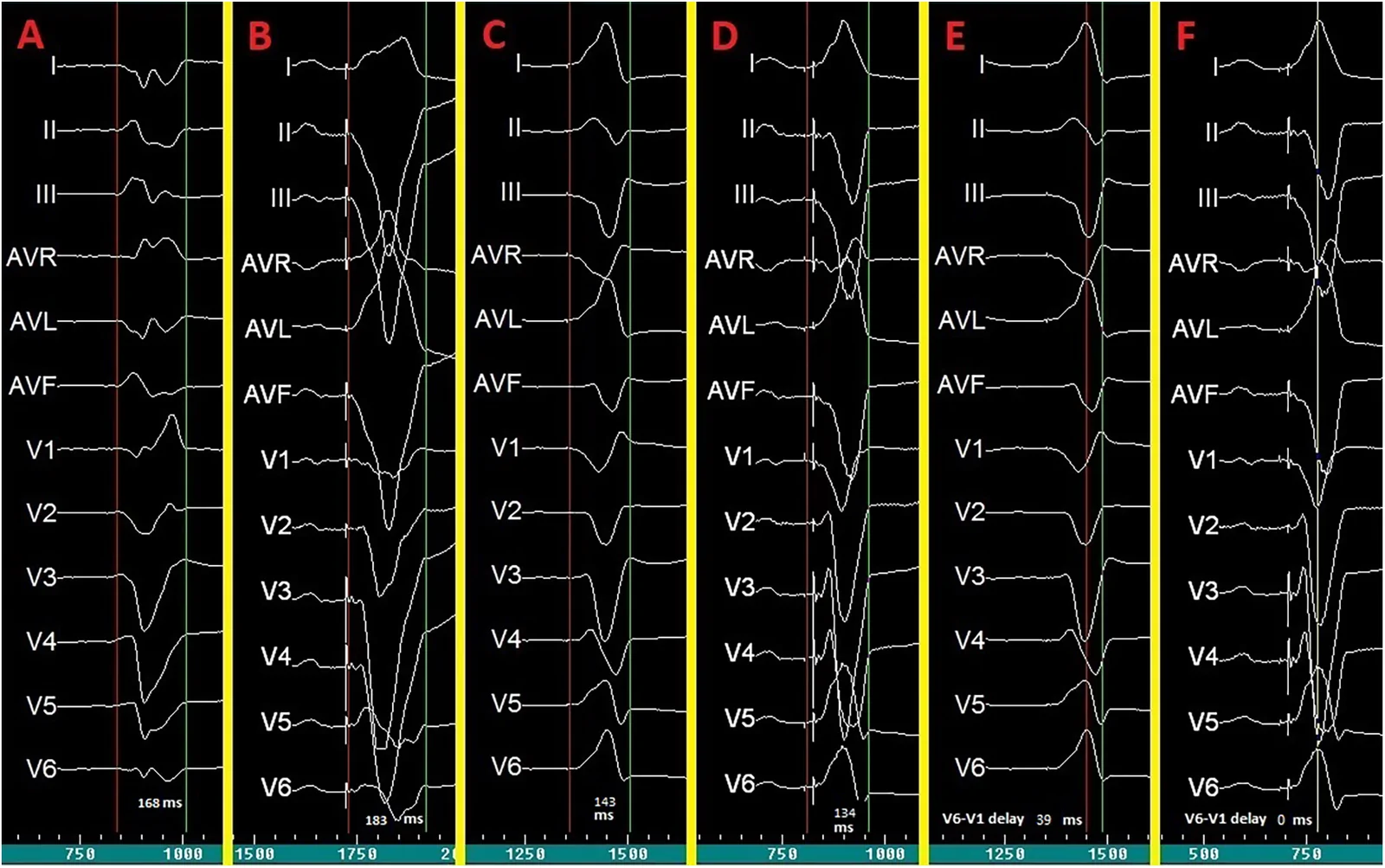
Pacing configuration testing and empirical ECG-guided interventricular programming. **(A)** Surface ECG tracing demonstrating the baseline QRS duration of 168 ms in the setting of right bundle branch block and high-grade atrioventricular block. **(B)** Surface ECG tracing during right ventricular pacing alone, demonstrating a paced QRS duration of 183 ms. **(C)** Surface ECG tracing during isolated deep septal pacing demonstrating QRS narrowing to 143 ms. **(D)** Surface ECG tracing during combined deep septal and right ventricular pacing with a septal pre-excitation of 20 ms, demonstrating further QRS narrowing to 134 ms. **(E)** Measurement of the V6-to-V1 R-wave peak time difference during isolated deep septal pacing, showing a V6-to-V1 R-wave peak time difference of 39 ms. **(F)** Measurement of the V6-to-V1 R-wave peak time difference during combined deep septal and right ventricular pacing, demonstrating near-equalization of interventricular activation timing. All paced QRS durations were measured from the pacing stimulus artifact to QRS offset.

At the two-week follow-up visit, before interventricular optimization, the patient had already reported partial improvement in dizziness and exertional symptoms. Device interrogation and pacing configuration testing were performed to further characterize ventricular activation patterns relative to the baseline electrocardiographic substrate (Figure 4A). Right ventricular pacing alone produced a paced QRS duration of 183 ms (Figure 4B). Isolated deep septal pacing reduced QRS duration to 143 ms and demonstrated a V6-to-V1 activation delay of 39 ms, indicating earlier left ventricular activation (Figures 4C,E). ECG-guided interventricular (VV) optimization was then performed using serial 12-lead surface ECG recordings across VV intervals ranging from −30 to +30 ms, with the septal lead programmed either before or after the right ventricular lead. All VV configurations were assessed using identical pacing outputs for both leads. QRS duration and V6-to-V1 activation timing were assessed under each configuration; LVEF was also assessed across the tested configurations and remained stable at 60%, without any clinically meaningful change between settings. The septal lead was ultimately programmed to pace 20 ms before the right ventricular lead; this configuration provided the narrowest observed paced QRS duration (134 ms) together with near-equalization of V6-to-V1 activation timing (Figures 4D,F), and was retained for chronic programming. The device was programmed in DDDR mode with a paced atrioventricular delay of 140 ms and a sensed atrioventricular delay of 110 ms, with both ventricular leads remaining active for biventricular pacing. Ventricular pacing minimization was intentionally not enabled because of the patient's persistent high-grade atrioventricular conduction disease and substantial baseline intraventricular conduction disturbance. At one-month follow-up, device interrogation demonstrated a biventricular pacing percentage of 96%, with atrial pacing of 5%. At the same visit, the patient demonstrated marked symptomatic improvement, with functional status improving from NYHA class III to class I–II and NT-proBNP decreasing to 369 pg/mL.

Follow-up transthoracic echocardiography at six months demonstrated preserved left ventricular systolic function (LVEF 60%) without LVOT obstruction at rest or with provocation. The septal lead position remained stable within the interventricular septum (Figure 3D), and trace tricuspid regurgitation remained unchanged from baseline. These echocardiographic findings remained unchanged at one year. At one-year follow-up, the patient was clinically well, with NYHA functional class maintained at I–II and NT-proBNP in the 200–400 pg/mL range. Device interrogation continued to demonstrate a high biventricular pacing percentage (98%), with stable lead electrical parameters and no device-related complications.

## Discussion

Management of patients with hypertrophic cardiomyopathy (HCM), preserved left ventricular ejection fraction (LVEF), symptomatic high-grade atrioventricular block, and high anticipated ventricular pacing burden remains insufficiently addressed by current pacing guidelines (2). Such patients require permanent ventricular pacing and may also have indications for implantable cardioverter-defibrillator therapy despite not fulfilling conventional criteria for cardiac resynchronization therapy, creating a clinically relevant gap in pacing strategy selection. Given the anticipated high ventricular pacing burden, conventional right ventricular pacing was considered undesirable because of its recognized potential to promote ventricular dyssynchrony in the structurally abnormal substrate of HCM (2). In this case, a CRT-D platform was implanted, with the lumenless septal lead connected to the LV port. This configuration subsequently permitted independent interventricular (VV) programming and empirical ECG-guided electrical optimization during follow-up.

Serial electrocardiographic assessment provided further insight into the electrical effects of incremental septal lead advancement. Progressive septal lead advancement was associated with stepwise shortening of the V6 R-wave peak time from 114 ms to 87 ms and paced QRS narrowing from 168 ms to 143 ms, consistent with progressive changes in ventricular activation during incremental lead advancement. The native electrocardiogram demonstrated a right bundle branch block pattern with fragmentation and notching across multiple leads, findings compatible with diffuse intraventricular conduction disturbance in the setting of severe myocardial hypertrophy rather than isolated right bundle branch block alone (4). These findings were obtained despite marked septal hypertrophy, which limited deeper lead advancement in the present case. Similar technical challenges have been described previously in patients with markedly hypertrophied interventricular septa (4, 5). Compared with the native ECG, paced QRS complexes exhibited a transition from a qR to a Qr configuration in lead V1, together with less conspicuous QRS fragmentation across multiple leads.

At the two-week follow-up visit, systematic pacing configuration testing was performed to guide device programming and characterize residual interventricular activation patterns. Right ventricular pacing alone produced a QRS duration of 183 ms, whereas isolated deep septal pacing reduced QRS duration to 143 ms with a V6-to-V1 activation delay of 39 ms, indicating that left ventricular activation preceded right ventricular activation. ECG-guided interventricular (VV) optimization, performed using serial 12-lead ECG recordings, identified septal pre-excitation of 20 ms relative to right ventricular pacing as the configuration that produced the narrowest paced QRS duration (134 ms) together with near-equalization of V6-to-V1 activation timing. These findings suggest that individualized interventricular timing adjustment can provide incremental electrical optimization beyond deep septal pacing alone by reducing residual right ventricular activation delay, irrespective of whether definitive left bundle branch capture has been achieved.

Recent evidence increasingly supports conduction system pacing as an alternative to conventional right ventricular pacing in patients with atrioventricular block and preserved left ventricular ejection fraction. A 2025 systematic review and meta-analysis by Ahsan et al., including 14 studies and 3,062 patients, found that left bundle branch area pacing was associated with shorter paced QRS duration, higher left ventricular ejection fraction, and fewer heart failure hospitalizations compared with right ventricular pacing, although the authors emphasized heterogeneity across studies and the need for more rigorous evidence (6). The LBBP-RESYNC trial provided randomized evidence that left bundle branch pacing-based CRT resulted in greater LVEF improvement than conventional biventricular CRT in patients with nonischemic cardiomyopathy and left bundle branch block (7). The ongoing Left vs Left randomized trial (NCT05650658) is further evaluating conduction system pacing versus biventricular pacing in patients with LVEF ≤50% and either a wide QRS or an anticipated pacing burden >40% (8). However, these studies do not specifically address patients with HCM, marked septal hypertrophy, preserved ejection fraction, and a concurrent indication for defibrillator therapy. In this setting, a particular challenge is how to address residual interventricular activation delay when deep septal pacing is achieved without definitive evidence of conduction-system capture.

This clinical combination also complicates device selection. Reports of patients requiring both ICD therapy and physiological pacing remain limited. One comparable case involved a patient with non-obstructive HCM, nonsustained ventricular tachycardia, and high anticipated pacing burden in whom LBBAP was attempted during ICD implantation; however, lead penetration failure resulted in left ventricular septal pacing (4). In the present case, a CRT-D platform was selected to provide defibrillation capability while allowing long-term ventricular pacing without conventional RV pacing. An ICD system incorporating a dedicated conduction-system pacing port could also be considered in principle; in the present configuration, however, independent septal and RV outputs and use of the LV port subsequently allowed interventricular timing to be modified according to the patient's individual electrical response.

Thus, the principal relevance of this case lies not in the choice of a particular pacing lead or in establishing a specific capture mechanism, but in demonstrating that individualized ECG-guided interventricular programming can provide additional electrical optimization after deep septal pacing. Whether this electrical improvement translates into incremental clinical benefit remains uncertain and requires further evaluation.

Clinical and biochemical improvement was observed during follow-up, including improvement in functional status from NYHA class III to class I–II and reduction of NT-proBNP from 1,868 to 369 pg/mL, with both remaining stable at one year. Importantly, the patient had already reported partial improvement in dizziness and exertional symptoms before the two-week interventricular optimization, indicating that the initial symptomatic improvement preceded the optimization procedure. The subsequent clinical and biochemical improvement was likely multifactorial. Restoration of atrioventricular synchrony and an adequate ventricular rate following correction of high-grade atrioventricular block, together with initiation of beta-blocker therapy, may each have contributed to the observed clinical response. The independent contribution of deep septal pacing and subsequent interventricular optimization cannot be disentangled from these factors in a single-patient case report.

The present findings are best interpreted as evidence of deep septal pacing with possible engagement of the left-sided conduction system rather than unequivocal confirmation of left bundle branch capture. Although stimulus-to-LVAT measurements were systematically assessed, no reproducible change suggestive of a transition between myocardial and conduction-system capture was observed during output testing, and no reproducible QRS morphology transition was documented. The paced ECGs demonstrated a relatively gradual initial QRS upstroke, suggesting a substantial myocardial activation component. A left bundle branch potential was not recorded. Accordingly, the available electrophysiological and electrocardiographic findings were insufficient to establish definitive conduction-system capture (3, 9).

The limitations of the present case should be acknowledged. Cardiac magnetic resonance imaging and genetic testing had reportedly been performed previously at an outside institution but were unavailable for review or inclusion in the present report. Their absence limited comprehensive phenotypic characterization but did not preclude interpretation of the pacing strategy and device programming described in this case. Finally, as a single case, these observations cannot establish causality or generalizability and should therefore be regarded as hypothesis-generating, requiring confirmation in additional clinical experience and future studies.

## Conclusion

In selected patients with hypertrophic cardiomyopathy, preserved ejection fraction, and high anticipated ventricular pacing burden, a CRT-D platform incorporating a septal pacing lead may provide flexibility for individualized interventricular electrical optimization when conventional right ventricular pacing is considered undesirable. In this case, ECG-guided interventricular programming reduced residual interventricular activation delay and provided additional electrical optimization beyond deep septal pacing alone; however, the clinical significance of this electrical improvement remains uncertain, as the observed clinical response was likely multifactorial. These findings suggest that systematic post-implant electrical assessment and individualized VV programming may represent an opportunity to further optimize ventricular activation in selected patients with complex structural and conduction substrates. Further studies are needed to determine whether this approach provides incremental clinical benefit.

## Data Availability

The original contributions presented in the study are included in the article/Supplementary Material, further inquiries can be directed to the corresponding author.

## References

[1] Arbelo E Protonotarios A Gimeno JR Arbustini E Barriales-Villa R Basso C. 2023 ESC guidelines for the management of cardiomyopathies. Eur Heart J. (2023) 44(37):3503–626. 10.1093/eurheartj/ehad194 37622657

[2] Glikson M Nielsen JC Kronborg MB Michowitz Y Auricchio A Barbash IM. 2021 ESC guidelines on cardiac pacing and cardiac resynchronization therapy. Eur Heart J. (2021) 42(35):3427–520. 10.1093/eurheartj/ehab364 34455430

[3] Huang W Chen X Su L Wu S Xia X Vijayaraman P. A beginner’s guide to permanent left bundle branch pacing. Heart Rhythm. (2019) 16(12):1791–6. 10.1016/j.hrthm.2019.06.016 31233818

[4] Zhu H Wang Z Li X Yao Y Huang W Liu Z. The initial experience of left bundle branch area pacing in patients with hypertrophic cardiomyopathy. Pacing Clin Electrophysiol. (2022) 45(9):1065–74. 10.1111/pace.14563 35895634

[5] Valappil SP Anand AB Ghosh A Subramanyan K Jaiswal P Jayanthi K. Left bundle branch area pacing in patients with severe interventricular septal hypertrophy: a multicenter study assessing feasibility, safety, and outcomes of lumenless and stylet-driven leads. Heart Rhythm O2. (2025) 6(5):588–97. 10.1016/j.hroo.2025.02.018 40496588 PMC12147595

[6] Ahsan I Hennawi HA Bedi A Khan MK Duseja N Ho RT. Left bundle branch area pacing versus right ventricular pacing in patients with atrioventricular block: a systematic review and meta-analysis. J Cardiovasc Electrophysiol. (2025) 36(2):501–11. 10.1111/jce.16548 39775893 PMC11837886

[7] Wang Y Zhu H Hou X Wang Z Zou F Qian Z. Randomized trial of left bundle branch vs biventricular pacing for cardiac resynchronization therapy. J Am Coll Cardiol. (2022) 80(13):1205–16. 10.1016/j.jacc.2022.07.019 36137670

[8] Chelu MG Ellenbogen KA. Left vs Left Randomized Clinical Trial. ClinicalTrials.gov Identifier: NCT05650658. Available online at: https://clinicaltrials.gov/study/NCT05650658 (Accessed 2026).

[9] Burri H Jastrzebski M Cano Ó Čurila K De Pooter J Huang W. EHRA Clinical consensus statement on conduction system pacing implantation: executive summary. Europace. (2023) 25(4):1237–48. 10.1093/europace/euad044 37061850 PMC10105857

